# Effects of arachidonic acid supplementation in maturation diet on female reproductive performance and larval quality of giant river prawn (*Macrobrachium rosenbergii*)

**DOI:** 10.7717/peerj.2735

**Published:** 2016-11-29

**Authors:** Chanpim Kangpanich, Jarunan Pratoomyot, Nisa Siranonthana, Wansuk Senanan

**Affiliations:** 1 Department of Fisheries, Faculty of Agriculture and Natural Resources, Rajamangala University of Technology Tawan-ok, Chonburi, Thailand; 2 Department of Aquatic Science, Faculty of Science, Burapha University, Chonburi, Thailand; 3 Institute of Marine Science, Burapha University, Chonburi, Thailand

**Keywords:** *Macrobrachium rosenbergii*, Giant river prawn, Arachidonic acid (ara), Maturation diet, Essential fatty acid

## Abstract

The giant river prawn (*Macrobrachium rosenbergii*) is one of the most farmed freshwater crustaceans in the world. Its global production has been stalling in the past decade due to the inconsistent quality of broodstock and hatchery-produced seeds. A better understanding of the role of nutrition in maturation diets will help overcome some of the production challenges. Arachidonic acid (20:4 n-6, ARA) is a fatty acid precursor of signaling molecules important for crustacean reproduction, prostaglandins E and F of the series II (PGE2 and PGF2*α*), and is often lacking in maturation diets of shrimp and prawns. We examined the effects of ARA in a combination of different fish oil (FO) and soybean oil (SO) blends on females’ reproductive performance and larval quality. Adult females (15.22 ± 0.13 g and 11.12 ± 0.09 cm) were fed six isonitrogenous and isolipidic diets containing one of two different base compositions (A or B), supplemented with one of three levels of *Mortierella alpine*-derived ARA (containing 40% active ARA): 0, 1 or 2% by ingredient weight. The two base diets differed in the percentages of (FO and SO with diet A containing 2% SO and 2% FO and diet B containing 2.5% SO and 1.5% FO, resulting in differences in proportional contents of dietary linoleic acid (18:2n-6, LOA) and docosahexaenoic acid (22:6n-3, DHA)). After the eight-week experiment, prawns fed diet B with 1 and 2% ARA supplement (B1 and B2) exhibited the highest gonadosomatic index (GSI), hepatosomatic index (HSI), egg clutch weight, fecundity, hatching rate, number of larvae, and reproductive effort compared to those fed other diets (*p* ≤ 0.05). Larvae from these two dietary treatments also had higher tolerance to low salinity (2 ppt). The maturation period was not significantly different among most treatments (*p* ≥ 0.05). ARA supplementation, regardless of the base diet, significantly improved GSI, HSI, egg clutch weight and fecundity. However, the diets with an enhanced ARA and LOA (B1 and B2) resulted in the best reproductive performance, egg hatchability and larval tolerance to low salinity. These dietary treatments also allow for effective accumulation of ARA and an n-3 lcPUFA, DHA in eggs and larvae.

## Introduction

The giant river prawn, *Macrobrachium rosenbergii*, is an economically important species cultured in the Indo-Pacific region (New, 2005). It is one of the most farmed freshwater crustaceans in the world, especially in the tropical and subtropical regions. World-leading producers of this species included China, Bangladesh and Thailand (117,402, 43,713 and 18,168 tons) in 2013 (FAO, 2016). Its global production has been stalling in the past decade due to inconsistent quality of broodstock and hatchery-produced seeds. Like other economically important crustaceans, a nutritionally balanced maturation diet is key to high-quality seed production. Even though the giant river prawn has been successfully domesticated for decades, seeds derived from hatcheries still vary in quantity and quality (Nhan et al., 2009).

Considerable knowledge on prawn nutrition has been generated during the past 20 years (New et al., 2009) but formulation diets can still be improved by using locally available material and supplementation with some overlooked nutrients. In crustaceans, reproductive functions like release of hatching factors (Holland et al., 1985) and ovarian maturation (Sarojini et al., 1989) are typically under the control of prostaglandins (PGs), a group of biologically active lipid compounds and also a subclass of eicosanoids. Prostaglandins are derived from long chain polyunsaturated fatty acids (C ≥ 20; lcPUFA), such as arachidonic acid (20:4n-6, ARA), eicosapentaenoic acid (20:5n-3, EPA) and dimo-gamma-linolenic acids (20:6n-3, DGLA) (Smith, DeWitt & Garavito, 2000). ARA is an important precursor of PGE2 and PGF2_*α*_. It is one of the fatty acids preferentially retained in the ovaries of crustaceans (Kumar et al., 2013). ARA in prawn maturation diets has tended to be overlooked in preference to EPA and DHA.

*M. rosenbergii* lacks the ability to synthesize linolenic acid (18:3n-3, LNA) and linoleic acid (18:2n-6, LOA) (D’Abramo & Sheen, 1993) and has limited ability to elongate and desaturate short-chain n-3 and n-6 polyunsaturated fatty acids (PUFA) (C18) to long chain PUFA (lcPUFA, C ≥ 20; Reigh & Stickney, 1989). In crustaceans, empirical evidence has suggested that ARA plays an important role in reproduction (e.g.,  Wouter et al., 2001; Glencross, 2009; Coman et al., 2011) and influences spawning performance, egg quality, embryonic and larval development, and larval quality (e.g., Millamena, 1993; Cahu, Cuzon & Quazuguel, 1995; Ravid et al., 1999; Meunpol, Meejing & Piyatiratitivorakul, 2005; Huang et al., 2008). In gravid female *M. rosenbergii*, the ovary and eggs also contain high ARA (approximately 2–3% of total fatty acids) (Tidwell et al., 1998; Cavalli et al., 2001; Balamurugan, Mariappan & Balasundaram, 2015), although much lower than in marine shrimp (7.1% for ARA in *Penaeus monodon* Huang et al., 2008). It is not clear whether *M. rosenbergi*i truly does not require as much lcPUFA as marine species, or whether its natural diets, derived mainly from freshwater systems, are lacking these fatty acids.

ARA is often inadequate in pelleted maturation diets because of the naturally low base level of ARA derived from fish oil (less than 0.1–1.6%, Turchini, Torstensen & Ng, 2009). Supplementation of ARA in diets even at a low level (7.5 mg g^−1^ diet ingredients) can enhance reproduction of tank-domesticated *P. monodon* (Coman et al., 2011). In addition to ARA, LOA and n-3 lcPUFA seem particularly important for *M. rosenbergii* reproduction (Cavalli, Lavens & Sorgeloos, 1999). Maturation diets containing a high level of LOA (approximately 13 mg g^−1^ DW) enhanced fecundity of females compared to those containing a low n-6 fatty acid level (approximately 4 mg g^−1^) (Cavalli, Lavens & Sorgeloos, 1999). Moreover, inclusion of high levels of both n-3 lcPUFA and n-6 PUFA in the spawner diets (15 mg g^−1^ and 13 mg g^−1^ DW, approximately 28–30% of total fatty acids) improved hatching rates and larval ammonia tolerance. For this current study, we built on this maturation diet and a diet formulation recommended by the Thai Department of Fisheries based on locally available diet ingredients (Somseub, 2009). Our previous study hinted at the importance of LOA, provided by soy oil and soy meal, in improved growth performance and possibly in reproduction. A mixture of 2% SO and 1% *Schizochytrium* sp. yielded the best growth performance in juvenile *M. rosenbergii* (unpublished data). Some individuals in this dietary treatment reached earlier maturation compared to the control fish oil (FO) diet.

The present study investigated the effect of ARA and LOA given adequate n-3 lcPUFA (FO 1.5–2% of total ingredients) on the reproductive performance of *M. rosenbergi*i female broodstock and larval quality, namely the tolerance to low salinity. We examined the effects of ARA supplementation in two base diets varying in percentages of FO and SO (resulting in different proportional contents of LOA and n-3 lcPUFA). We hypothesized that increased dietary ARA along with a higher LOA level would improve reproductive performance of female *M. rosenbergi*i. Our results provided insights to the understanding of these fatty acids in facilitating the prawn maturation process, and in larval development. Our findings also help fine tune the formulation of optimal maturation diets based on locally available material that will improve larval quality.

## Material and Methods

### Experimental design and prawns

We used a 6 × 3 completely randomized design (CRD) in this experiment, with six treatments performed in triplicate. Each experimental unit (a cage)consisted of a group of 15 females receiving each of the six diet formulations. Each cage was sub-divided into fifteen 18 × 13 × 12 cm compartments, each of which held one individual female. To have a manageable number of individuals per tank, to ensure optimal water quality for growth and reproduction, and to have an adequate number of individuals for all analyses, we created the entire experimental setting in two 3 × 3 × 1.2 m recirculating concrete tanks. Each tank contained 270 individual female prawns. Individuals raised in one tank were used for the analyses of females’ reproductive performance and fatty acids of eggs and larvae. Those raised in the second tank were used for determining fatty acid compositions in muscle and stage II–V ovaries, as well as for estimating Gonadosomatic index (GSI) and Hepatosomatic index (HSI). Because the hepatopancreas is a major lipid storage organ in crustaceans, HSI is an indication of the status of lipid and nutrient storage. The experiment lasted eight weeks.

Approximately four-month-old *M. rosenbergii* adults were obtained from a commercial prawn farm in Chachoengchao Province, Thailand. Individuals were acclimatized to 28 °C in a freshwater recirculation system at Rajamangala University of Technology Tawan-ok and fed a (commercial prawn 40% protein and 8% lipid) diet for seven days.

At the start of the experiment, females and males were randomly selected and stocked into the experimental set-ups. The initial weights and total lengths of females and males were 15.22 ± 0.13 g and 11.12 ± 0.09 cm, and 17.68 ± 0.22 g and 11.44 ± 0.09 cm, respectively. Males and females were kept separately until mating. The males used in the experiments were raised together in another two 1 × 3 × 1 m communal concrete tanks, each containing 250 individuals. Only females received the experimental diets; the males were fed a commercially formulated diet (Charoen Pokphand Foods, 38% protein and 8% lipid). Experimental animals were fed twice daily (07.00 and 18.00) at approximately 5% of body weight.

Animals were maintained at an optimal water quality where the water temperature during the experimental trial ranged from 26.4–28.3 °C, the dissolved oxygen from 7.12–7.78 mg L^−1^, pH from 8.19–8.38, alkalinity from 178.42–242.33 mg L^−1^, hardness from 129.42–146.33 mg L^−1^. Nitrogenous compounds, namely ammonia nitrogen, NO_2_-N and NO_3_-N were 0.01–0.06, 0.01–0.04 and 0.05–0.08 mg L^−1^, respectively. Temperature, pH (YSI, Model 63) and dissolved oxygen were monitored daily. Alkalinity (titration method detailed in (American Public Health Association (APHA), 1980), hardness, ammonia (indophenol blue method detailed in Grasshoff, 1976) and nitrate (the diaxotization method provided in Grasshoff, 1976) were determined on a weekly basis. The water quality parameters were consistent across treatments.

### Experimental diets

Experimental diets were formulated to meet the nutritional requirements of adult *M. rosenbergii* as recommended by Somseub (2009) and Cavalli, Lavens & Sorgeloos (1999) (Table 1). The six experimental isonitrogenous and isolipidic diets contained approximately 42% protein and 9% lipid by weight. Each diet contained one of the two base compositions (A or B) and varying ARA supplementation (0, 0.4 or 0.8% by ingredient weight). The ARA used in this study was derived from *Mortierella alpine*, containing 40% ARA. One of the base diets (base diet A) contained 2% FO and 2% SO by ingredient weight (A0, A1, A2, Table 2) and the other (base diet B) contained 1.5% FO and 2.5% SO (diets B0, B1, B2). For the best reproductive performance of *M. rosenbergii* broodstock, the experimental HH diet in Cavalli, Lavens & Sorgeloos (1999) contained 2% FO and 2% corn oil (CO) resulting in 15 and 13 mg g^−1^ DW of n-3 and n-6 PUFA, respectively. We substituted SO for CO because SO is cheaper and more readily available in Thailand. Based on our preliminary fatty acid analysis, the fatty acid profiles of both oil types were similar; they contained similar saturates, monoenes and LOA contents (51% and 54% LOA of total fatty acids for SO and CO, respectively). Compared to the HH diet, base diet A had a comparable DHA content while diet B had comparable LOA contents (Table 2). Each base diet was supplemented with one of three levels of ARA, either 0, 0.4 or 0.8% by ingredient weight. All experimental diets were subjected to chemical composition and fatty acid analysis. All analyses were performed in triplicate per treatment.

**Table 1:** Ingredients (% dry weight) and proximate composition (%) of the six experimental diets.

Ingredients	A0	A1	A2	B0	B1	B2
	2%FO+2%SO (Base diet A)			1.5%FO+2.5%SO (Base diet B)		
	0%ARA	0.4%ARA	0.8%ARA	0%ARA	0.4%ARA	0.8%ARA
Fish meal^b^	35.0	35.0	35.0	35.0	35.0	35.0
Soybean meal	25.0	25.0	25.0	25.0	25.0	25.0
Shrimp shell meal^c^	14.0	14.0	14.0	14.0	14.0	14.0
Corn grain^d^	5.0	5.0	5.0	5.0	5.0	5.0
Wheat meal	5.0	4.0	3.0	5.0	4.0	3.0
Rice bran	10.0	10.0	10.0	10.0	10.0	10.0
Fish oil	2.0	2.0	2.0	1.50	1.50	1.50
Soy oil	2.0	2.0	2.0	2.50	2.50	2.50
ARA^e^	0	0.4	0.8	0	0.4	0.8
Binder^f^	1.0	1.0	1.0	1.0	1.0	1.0
Vitamin and mineral premix^g^	1.0	1.0	1.0	1.0	1.0	1.0
**Proximate composition**						
Moisture	6.74	6.88	5.77	6.09	5.86	6.17
Protein	42.19	42.60	41.70	41.53	42.53	42.04
Lipid	9.41	9.36	9.17	9.27	9.47	9.65
Ash	16.70	16.90	17.73	16.78	16.21	15.95
^a^NFE	24.96	24.26	25.63	26.33	25.93	26.19
Digestible energy						
(Kcal 100 g^−1^)	321.32	319.17	318.50	320.89	324.88	324.82

**Notes.**

a NFE=nitrogen free extract + fiber.

b Mix of marine fish containing 55% protein from Siam fish meal Lp. Thailand.

c Shrimp shell meal or shrimp meal contain heads and shells of marine shrimps (1 mm) from Thailand.

d Corn grain or corn meal is made by grinding dried corn kernels (1 mm) from Thailand.

e Arachidonic acid 40% made from *Mortierella alpine* (single cell oil). from Anhui Minmetals development I/E Co., Ltd. China.

f 5*α*-starch from Mario bio products., Co. Ltd. Thailand.

g Vitamin and mineral premix : retinoplamitate (A), 5,000 IU kg^−1^; chlolecalciferol (D_3_), 1,000 IU kg^−1^; *α*-tocopherol, 100 IU kg^−1^; menadione (K), 1.0%; thiamine (B1) 0.3%; riboflavin (B2) 1.4%; pyridonine (B6) 0.9%; cyanocoblamine (B12) 0.1%; panthothenic acid 3.6%; folic acid 0.4%; inositol 30%; biotin 0.1%; Se 0.1%; Fe 24%, Zn 38.4%, Cu 2.4%. from Thailand.

**Table 2:** Fatty acid compositions (% of total fatty acids) of the six experimental diets.

Fatty acids	A0	A1	A2	B0	B1	B2
	2%FO+2%SO (Base diet A)			1.5%FO+2.5%SO (Base diet B)		
	0%ARA	0.4%ARA	0.8%ARA	0%ARA	0.4%ARA	0.8%ARA
16:0	20.91 ± 1.16	18.04 ± 1.97	17.21 ± 0.91	17.44 ± 1.06	16.89 ± 1.19	16.59 ± 0.32
18:0	6.75 ± 0.31	6.42 ± 0.32	5.77 ± 0.11	5.49 ± 0.16	4.16 ± 0.21	4.35 ± 0.09
18:1n-9	13.24 ± 0.24	12.24 ± 1.79	16.01 ± 1.05	16.34 ± 0.83	16.28 ± 0.88	15.33 ± 0.33
18:2n6(LOA)	17.23 ± 0.96	14.85 ± 1.15	13.35 ± 0.74	20.39 ± 1.45	21.78 ± 0.38	22.49 ± 0.55
20:4n6(ARA)	2.77 ± 0.05	6.17 ± 0.26	9.43 ± 0.28	3.08 ± 0.08	5.74 ± 0.12	8.33 ± 0.04
18:3n3(LNA)	6.27 ± 1.93	7.66 ± 1.03	6.77 ± 0.83	7.82 ± 0.56	6.89 ± 0.38	7.31 ± 0.18
20:5n3(EPA)	4.54 ± 0.17	5.09 ± 0.16	5.58 ± 0.11	4.74 ± 0.04	4.43 ± 0.13	4.05 ± 0.09
22:6n3(DHA)	10.47 ± 0.26	9.99 ± 0.45	9.15 ± 0.05	7.41 ± 1.50	6.12 ± 0.30	6.78 ± 0.63
∑saturates	36.46 ± 1.33	34.47 ± 0.38	32.01 ± 1.52	31.59 ± 0.88	30.01 ± 0.37	28.13 ± 0.32
∑monoenes	22.27 ± 1.88	21.77 ± 1.01	24.37 ± 0.54	24.97 ± 0.48	25.03 ± 0.62	22.91 ± 0.45
∑n-6PUFA	19.99 ± 0.93	21.02 ± 1.06	22.77 ± 1.03	23.47 ± 1.41	27.52 ± 0.50	30.82 ± 0.54
∑n-3PUFA	21.27 ± 1.02	22.74 ± 0.86	21.51 ± 0.89	19.97 ± 2.05	17.44 ± 0.24	18.14 ± 0.61
∑n-3lcPUFA	15.01 ± 0.40	15.08 ± 0.60	14.73 ± 0.10	12.15 ± 1.49	10.56 ± 0.25	10.83 ± 0.71
∑n-3/∑n-6	1.06 ± 0.08	1.09 ± 0.08	0.95 ± 0.08	0.85 ± 0.13	0.64 ± 0.02	0.59 ± 0.03

**Notes.**

Values are given as the mean ± standard deviation (*n* = 3). Differing superscript letters highlight significant differences at *p* ≤ 0.05.

∑saturates = 14:0, 15:0, 16:0, 17:0, 18:0.

∑monoenes = 16:1, 17:1, 18:1n9.

∑n-6PUFA = 18:2n-6, 20:4n-6.

∑n-3PUFA = 18:3n-3, 20:5n-3, 22:6-n-3.

∑n-3lcPUFA = 20:5n-3, 22:6-n-3.

### Chemical composition of the experimental diets

We performed proximate analysis to determine diet moisture, crude protein, crude lipid, ash content and nitrogen-free extract (NFE) using standard procedures (AOAC, 2005). Moisture was measured a weight loss after oven drying at 105 °C for 12 h. Ash was determined by muffle furnace at 600 °C for 5 h. Crude protein was determined by the Kjeldahl method and lipid was determined by Fosslet fat analysis. The digestible energy of the experimental diets was calculated from standard physiological fuel values of 4 Kcal g^−1^ for crude protein and nitrogen-free extract and 9 Kcal g^−1^ for lipids (Cavalli, Lavens & Sorgeloos, 1999).

### Experimental sampling protocol

For female reproductive performance, we determined gonadosomatic index (GSI), hepatosomatic index (HSI), incubation period (i.e., from newly fertilized orange eggs, OE, to hatching), fecundity, reproductive effort, hatching rate, larval length and larval tolerance to low salinity. For fatty acid analysis, we determined fatty acid profiles of muscle tissue (only from gravid females), stage II–V ovaries, two stages of eggs (OE and BE stages) and larvae. For all analyses, we collected the data in triplicate and nine individuals per treatment were analyzed. With the exception of fatty acid analysis, all other analyses obtained nine independent values per replication. For fatty acid analysis of all tissue types, we prepared a homogenate for each replication from a pool of three individuals; only three values per treatment were obtained. We started to observe stage II ovaries at approximately one month after beginning the experiment. Stages III and IV developed one to two weeks after the prior stage. By the end of the experiment, most experimental animals developed stage V ovaries. The tissue samples were kept at −40 °C for fatty acid analysis.

### Lipid extraction and fatty acid analysis of diets and tissues

Total lipids from experimental diets and various tissues were extracted by homogenizing each sample in 20 ml ice-cold chloroform:methanol (2:1, v/v) containing 0.1% butylated hydroxytoluene for 20 min before the liquid fraction was transferred to a separating funnel. The residual matter was then subjected to a second round of extraction, after which the liquid portion was transferred to a separating funnel. To separate the non-lipid phase, 0.88% (w/w) KCl (approx. 25% of the total sample volume) was then added to the separating funnel, agitated to mix the contents, and then left until the solution separated into two layers. Total lipid was obtained by filtering the lower layer through anhydrous sodium sulfate before evaporating the collected fraction (Folch, Lees & Sloane-Stanley, 1957). Fatty acid methyl esters (FAMEs) were prepared from the total lipid by subjecting samples to acid-catalyzed transesterification by adding 1% sulphuric acid and then incubating them at 50 °C for 16 h (Christie, 1993). Gas-liquid chromatography (GC7820A; Agilent Technologies, Santa Clara, CA, USA) was then used to determine the FAME, with individual FAMEs being identified by comparison to known standards (Supelco 37-Component FAME Mix, Supelco, USA). The FAMEs were split-injected through a wall-coated capillary column (HP-Innowax column, 30 m × 0.25 mm id, 0.25 µm film thickness (Agilent J&W) and detected via a flame ionization detector (FID). Helium gas was used as the carrier at a constant flow rate of 1.1 ml min^−1^. The temperature program used was an initial 150 °C for 0.5 min, increasing to 170 °C at a rate of 5 °C min^−1^, held at 170 °C for 10 min, then increasing to 190 °C at a rate of 3 °C min^−1^, and then held at 190 °C for 28 min. Temperatures at the injection and detection ports were 230 °C and 250 °C, respectively. The fatty acid analyses were performed at the Institute of Marine Science, Burapha University, Thailand.

### Determination of ovarian development

**Figure 1:**
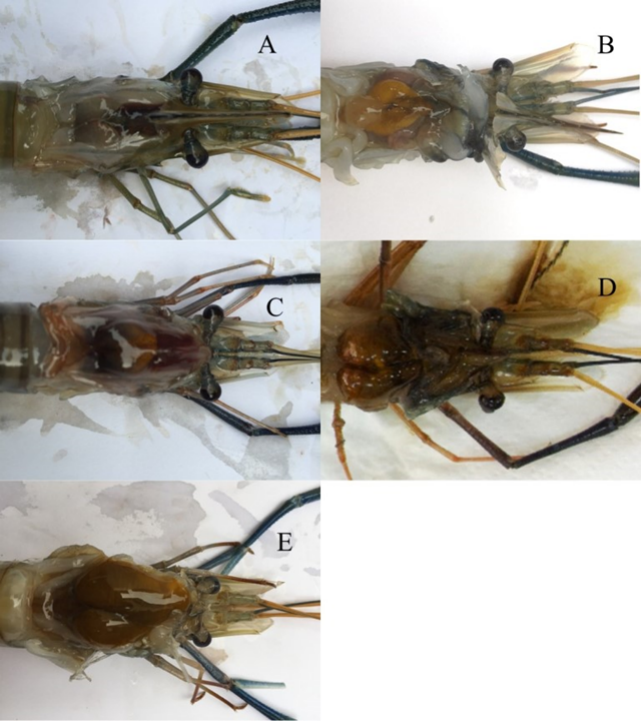
Descriptions of ovarian maturation stages of *M. rosenbergii* based on the criteria described in Damrongphol, Eangchuan & Poolsanguan (1991), Chang & Shih (1995) and Revathi et al. (2012). (A) Spent stage (Stage I). No ovarian tissue can be observed; this absence is characteristic of both non- developed and spent females. (B) Early previtellogenic stage (Stage II). The ovarian tissue is present as a small, yellow spot near the posterior part of the carapace. (C) Late previtellogenic stage (Stage III). Ovary, orange in color, can be observed from the posterior part of the carapace to the area just in front of the epigastric tooth. (D) Early vitellogenic stage (Stage IV). The orange ovarian tissues have grown and extended to the area of the epigastric tooth. (E) Late vitellogenic stage (Stage V). The ovarian tissues have extended to the anterior part of the carapace.

Molting and ovarian development of each female were monitored daily. Maturation stages of the ovary were classified into one of five stages, based on the ovarian size and color observed through the carapace, following the criteria detailed in Damrongphol, Eangchuan & Poolsanguan (1991), Chang & Shih (1995) and Revathi et al. (2012) (Fig. 1). There are five recognizable maturation stages to the ovary, i.e., stages I, II, III, IV and V. For each of nine gravid females per treatment, we estimated GSI and HSI based on the proportion of weight of a stage V ovary or a hepatopancreas to total body weight at the end of the experiment.

### Mating and determination of egg development

We paired a mature female to a male at a 1:1 ratio; the entire experiment required a total of 270 mating pairs. Each gravid female (with a stage V ovary) was mated in a compartment to a mature male immediately after molting. We manually placed a mature male, kept in one of the ‘male’ tanks (see above), in the compartment. After the eggs were fertilized (about 6–8 h after mating), the male was removed from the compartment to another tank without returning to the original male tank; each male was used only once. Approximately 8 h after mating, freshly fertilized eggs migrated to the female’s brood chamber in the abdominal area and became visible.

The female prawns in individual compartments were observed daily to visually determine embryonic development stage, based on the egg color (Habashy, Sharshar & Hassan, 2012). As embryonic stages advance, the egg color changes from orange (newly fertilized eggs, OE) to pale brown (mid-embryonic stage with a pair of eye spots on the yolk area becoming visible, BE) and deep brown (advanced embryos). We recorded the incubation period from the OE stage until hatching (days).

### Evaluation of fecundity and hatching rate

A 10-day-old egg clutch (brownish) (after OE become apparent), scraped from the brood chamber of each of three females per treatment was weighed to the nearest 0.01 g after removing any excess water by repeated blotting. Three independent samples of equal weight (0.2 g) from each clutch were counted under a microscope to estimate the total number of eggs per clutch. The averaged egg numbers did not differ statistically among treatments. We therefore extrapolated the averaged egg number per 0.2 g of 1,450 ± 72.13 eggs to an actual egg clutch weight for fecundity estimates for all treatments.

An egg clutch weight was the difference between a gravid female’s weight before and after hatching. Gravid females bearing brownish eggs were weighed and transferred into an aerated 20-L tank containing 14 ppt water. After spawning, the females were weighed and transferred back into the original compartment, while the spawned eggs remained in the hatching tank and then were used for the subsequent experimental steps. Fecundity was estimated for each individual female as the total number of eggs (actual clutch weight*(1,450 eggs/0.2 g)) per body weight (eggs/g BW female). The reproductive effort for each individual female was estimated as the percentage of egg weight to body weight. We estimated the number of newly hatched larvae to a female’s weight (number of larvae/g female) by counting the number of individuals in a sample of 100 ml water from a well-mixed tank and then extrapolating to 20 L. Hatching rates were determined from the number of larvae per total number of brownish eggs.

### Determination of larval quality: low-salinity tolerance

We tested the tolerance to low salinity of newly hatched larvae of the experimental females. The larvae from each treatment were exposed to a low salinity level of 2 ppt for 24 h. Each experimental unit contained 30 individual larvae. A group raised in 14 ppt water were treated as a control. At the end of the test, we recorded the survival for each treatment.

### Statistical analysis

We determined the differences in female reproductive performance, larval quality and fatty acid compositions in diets and various tissues among dietary treatments using one-way analysis of variance (ANOVA). The significance of the differences in means was determined by Duncan’s new multiple range test at a *p* value < 0.05. All statistical analyses were executed with SPSS version 17 for Windows (SPSS, Inc.). For the data (e.g., GSI, hatching rates) that were not normally distributed, we performed either square root arcsine or arcsine transformation before ANOVA. However, only non-transformed means are presented in the table.

To examine the variation of fatty acid profiles among various tissue types and treatments, we analyzed principal components from seven quantitative variables: percentages of five major fatty acids and total values of two fatty acid classes detected in our study (LOA, LNA, ARA, EPA, DHA, total saturates and total monoenes). Total n3-lcPUFA was treated as a supplementary variable on a principal component analysis (PCA) biplot for a better understanding of the ordination based on the seven active quantitative variables; it did not interfere with the ordination. This multivariate approach utilized all information available for each individual while at the same time reduced the dimensionalities of the data (from seven dimensions to two dimensions in our case). The first few principal components typically captured most of the variation in the data set. PCA was performed using algorithms implemented in the FactoMineR package (Lê, Josse & Husson 2008) and the biplot was created using factoextra package (Kassambara, 2015), developed in the R statistical language (R Core Team, 2015).

## Results

### Proximate compositions and fatty acid contents of the experimental diets

The proximate compositions of the experimental diets were similar (Table 1). Each experimental diet contained approximately 42% protein and 9% lipid while the ash and NFE content of each diet were approximately 17% and 26%, respectively. The digestible energy content of each diet was between 3,200 and 3,250 Kcal Kg^−1^.

The fatty acid composition of each diet generally reflects the level of ARA supplementation and different proportions of lipid sources in two base compositions (A and B) (Table 2). The proportional content of ARA detected in diets increased with the increasing proportions of ARA added to the ingredients. The percentage of ARA was highest in the A2 diet, containing 2% FO, 2% SO and 0.8% ARA of diet ingredients. The two base compositions clearly differed in the proportions of LOA, total n-3 lcPUFA and total saturates (Table 2). The base diet A, containing 2% SO and 2% FO, had higher total saturates and total n-3 lcPUFA than base diet B, containing 2.5% SO and 1.5% FO. DHA was particularly high in base diet A. Base diet B, on the other hand, had higher LOA than base A. The levels of total n-6 PUFA in most diets were higher than those of total n-3 lcPUFA. Ratios of total n-3 PUFA to total n-6 PUFA in all diets ranged from 0.59 (B2) to 1.06 (A0).

For n-6 PUFA, all diets contained LOA as a major component (averaged% of total fatty acid = 13.35–22.49%) with B2 containing the highest level of total n-6 PUFA and A0 containing the lowest level. For total n-3 PUFA, LNA, EPA and DHA were primary components. All diets contained approximately 6–7% LNA, 4–5% EPA and 6–10% DHA of total fatty acids. The total n-3 PUFA and n-3 lcPUFA were highest in diet A1. In diets A0-A3, LNA contents were slightly higher than EPA but lower than DHA contents. In contrast, LNA contents were higher than DHA in diets B0–B2. In all diets, dietary EPA levels were lower than DHA; EPA contents were similar among all diets (4.05–5.58% of total fatty acids). EPA to DHA ratios were similar among experimental diets, approximately 0.5 (0.43–0.72).

**Table 3:** Reproductive performance and larval quality of *M. rosenbergii* fed different experimental diets.

Parameter	Experimental diets					
	A0	A1	A2	B0	B1	B2
	2%FO+2%SO (Base diet A)			1.5%FO+2.5%SO (Base diet B)		
	0%ARA	0.4%ARA	0.8%ARA	0%ARA	0.4%ARA	0.8%ARA
GSI (%)	6.99 ± 0.82^c^	7.31 ± 0.41^bc^	7.88 ± 0.14^b^	6.05 ± 1.32^d^	8.59 ± 0.53^a^	8.73 ± 0.51^a^
HSI (%)	2.99 ± 0.44^c^	3.81 ± 0.34^b^	4.05 ± 0.42^b^	3.09 ± 0.95^c^	4.61 ± 0.57^a^	4.85 ± 0.43^a^
Egg clutch weight (g)	2.20 ± 0.36^c^	2.50 ± 0.24^b^	2.49 ± 0.22^b^	2.20 ± 0.18^c^	2.89 ± 0.38^a^	2.91 ± 0.11^a^
Fecundity (egg/g female)	1087.47 ± 164.16^c^	1191.15 ± 73.29^b^	1268.33 ± 128.62^b^	1136.57 ± 115.77^c^	1399.82 ± 187.96^a^	1354.62 ± 129.93^a^
No larval/g female	655.05 ± 81.64^d^	816.88 ± 80.80^bc^	852.19 ± 90.71^b^	731.54 ± 150.09^cd^	1094.48 ± 98.68^a^	1104.57 ± 68.52^a^
Hatching rate (%)	70.44 ± 12.76^c^	78.71 ± 11.02^c^	78.00 ± 9.65^bc^	74.87 ± 14.4^c^	90.39 ± 12.02^ab^	91.04 ± 4.26^a^
Reproductive effort (g egg/g female × 100)	13.06 ± 1.83^d^	14.38 ± 0.66^bc^	15.13 ± 1.20^b^	13.49 ± 1.01^cd^	16.61 ± 1.78^a^	16.79 ± 1.03^a^
Egg incubation period (days)	20.33 ± 1.22	20.22 ± 1.78	19.67 ± 1.58	20.78 ± 1.48	19.33 ± 1.32	19.96 ± 1.60
Larval length (mm)	1.92 ± 0.01	1.92 ± 0.01	1.91 ± 0.01	1.91 ± 0.01	1.90 ± 0.06	1.91 ± 0.01
Larval survival at 2 ppt	62.22 ± 9.43^b^	65.93 ± 16.39^b^	62.22 ± 6.45^b^	56.67 ± 14.43^b^	79.23 ± 4.65^a^	87.04 ± 3.89^a^
Larval survival at 14 ppt	100	100	100	100	100	100

**Notes.**

TITLE GSI Gonadosomatic index HSI hepatosomatic index OE newly fertilized, orange eggs

Values are given as the mean ± standard deviation (*n* = 9). Differing superscript letters (a–d) highlight significant differences at *p* ≤ 0.05.

### Reproductive performance of female prawns and larval quality

Females fed diets containing 0.4% and 0.8% ARA supplementation (A1, A2, B1and B2) had higher reproductive performance than those fed on diets without ARA addition (A0 and B0) (*p* < 0.05; Table 3). However, groups consuming enhanced ARA and LOA (B1 and B2) exhibited highest GSI, HSI, egg clutch weight, fecundity, hatching rate and reproductive effort (*p* < 0.05). Females fed diets B1 and B2 had GSI of 8.59 ± 0.53 and 8.73 ± 0.51% and HSI of 4.61 ± 0.57 and 4.85 ± 0.43%, respectively. The average egg clutch weights for these groups were 2.89 ± 0.38 and 2.91 ± 0.11 g. Their average fecundities were 1,399.82 ± 187.96 and 1,354.62 ± 129.93 eggs per female; reproductive effort was 16.61 ± 1.78 and 16.79 ± 1.03 g of eggs per g BW female. Hatching rates were higher than 90% for these groups. Female fed diets A1 and A2 had HSI of 3.81 ± 0.34 and 4.05 ± 0.42%. The average egg clutch weights for these groups were 2.50 ± 0.24 and 2.49 ± 0.22 g, average fecundities were 1,191.15 ± 73.29 and 1,268.33 ± 128.62 eggs per female, for A1 and A2, respectively. Larval lengths and egg incubation periods (OE to hatching) of all dietary treatments were not statistically different.

Larvae from females fed diets B1 and B2 were also more tolerant to low salinity compared to other dietary treatments (Table 3). At extremely low salinity (2 ppt), the survival at 24 h for these groups was higher than 79% (79.23 ± 4.65 and 87.04 ± 3.89% for offspring of females fed diets B1 and B2, respectively). Survival among all groups was not significantly different at 14 ppt (control).

### Fatty acid composition of the tissues

**Figure 2:**
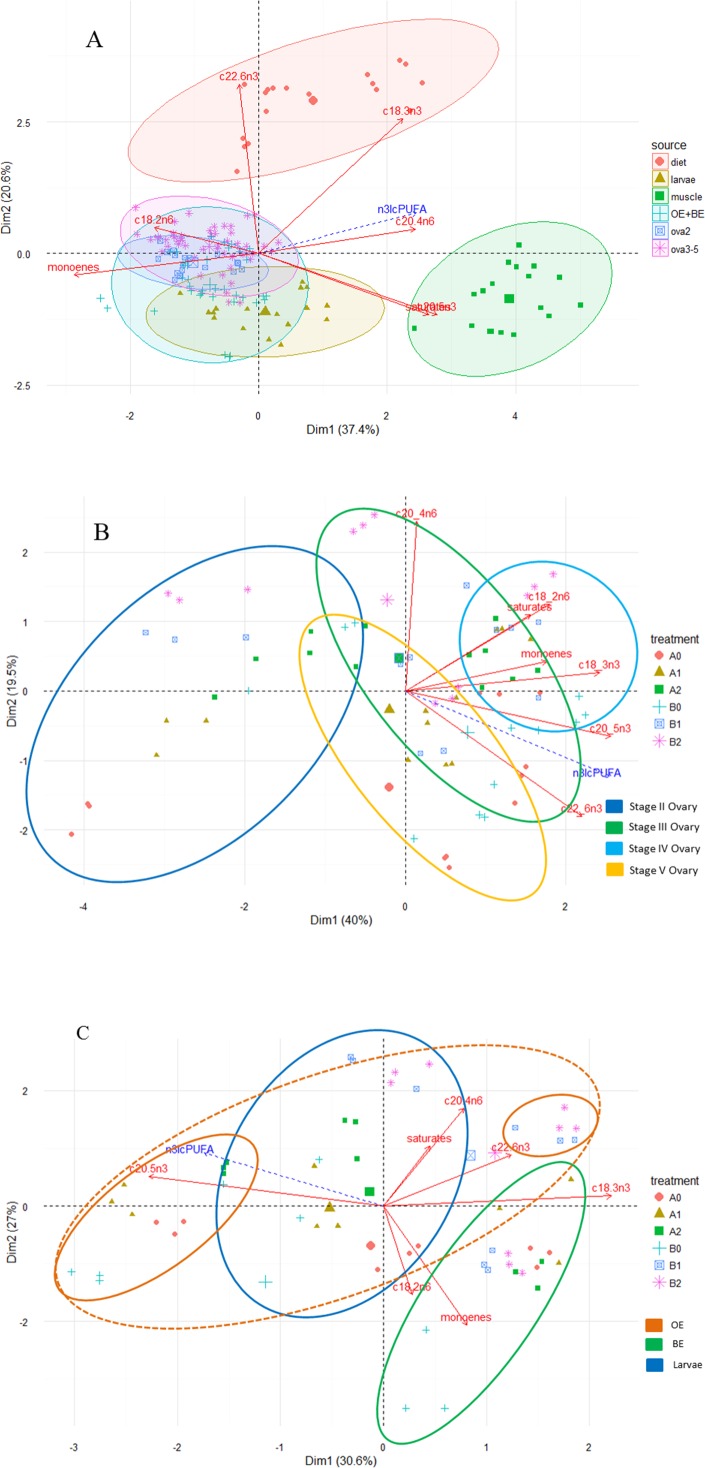
Principal component analysis (PCA) biplot of fatty acid compositions (% of total fatty acid) in diet and tissues of *M. rosenbergii*. (A) PCA ordination of fatty acid compositions in experimental diets and *M. rosenbergii* tissues, muscle, reproductive tissues and larvae. (B) Among-treatment variation of fatty acid compositions in ovaries II–V and (C) in eggs and larvae. OE is newly fertilized, orange eggs of *M. rosenbergii* and BE is mid-stage embryo, brown eggs. Each oval outline color represents a tissue type.

Principal component analysis revealed distinct fatty acid profiles based on diet and tissue type (Fig. 2A). The first two components explained 58% of total variation, with the first and second dimensions explaining 37.4% and 20.6%. Higher proportional contents of LNA, EPA and DHA in the fatty acid profiles separated diets from the tissues (dimensions 1, 2) and those of saturates, ARA and EPA separated muscle from the remaining tissues (dimension 1). Although fatty acid profiles of ovaries, eggs and larvae appeared similar, the PCA further illustrated variation in fatty acid profiles within and among tissue types as well as among treatment groups Figs. 2B and 2C.

Total saturates was the dominant type of fatty acid in all tissue types with some small fluctuations depending on the tissue and developmental stage (Tables 4–6). Overall, total n-6 PUFA in tissues was higher than total n-3 PUFA and total n-3 lcPUFA levels. For PUFA, total n-6 PUFA contents were high in all tissue types (approximately 19–28% of total fatty acids) and total n-3 PUFA was higher in muscle (>15% of total fatty acids) than in other tissue types (<15%). LOA contents were consistently high in all tissue types, while the n-3 lcPUFA, namely EPA and DHA, fluctuated upon the base diet and tissue type. ARA in tissues reflected the diet supplementation levels although patterns of retention in muscle and ovaries were different. LNA and EPA were the dominant n-3 PUFA in muscle tissue whereas DHA contributed to a larger percentage of total fatty acids in ovarian, egg and larval tissues. LNA was substantially reduced in ovarian, egg and larval tissues (<2%). For most fatty acids, egg and larval fatty acid contents were similar to those of mature ovaries (stage V).

**Table 4:** Fatty acid compositions (% of total fatty acids) of the muscle tissue of mature female *M. rosenbergii* fed different experimental diets.

Fatty acids	A0	A1	A2	B0	B1	B2
	2%FO+2%SO (Base diet A)			1.5%FO+2.5%SO (Base diet B)		
	0%ARA	0.4%ARA	0.8%ARA	0%ARA	0.4%ARA	0.8%ARA
16:0	22.27 ± 1.64	21.97 ± 1.45	22.54 ± 0.02	23.67 ± 0.11	21.95 ± 0.92	22.46 ± 0.63
18:0	8.40 ± 0.74^ab^	9.02 ± 0.32^a^	8.92 ± 0.59^a^	8.00 ± 0.21^b^	8.25 ± 0.40^ab^	8.16 ± 0.36^ab^
18:1n-9	13.73 ± 0.44^a^	12.13 ± 0.97^ab^	11.90 ± 0.79^b^	11.63 ± 0.38^b^	10.51 ± 0.31^b^	10.42 ± 1.94^b^
18:2n6 (LOA)	14.22 ± 1.22^c^	14.38 ± 1.13^c^	16.48 ± 0.54^b^	18.63 ± 0.69^a^	18.59 ± 0.91^a^	18.85 ± 1.26^a^
20:4n6 (ARA)	5.46 ± 0.70^c^	7.98 ± 0.85^b^	10.31 ± 0.86^a^	5.39 ± 0.66^c^	7.74 ± 0.38^b^	8.44 ± 0.52^b^
18:3n3 (LNA)	4.97 ± 1.93	4.49 ± 1.63	3.54 ± 1.56	4.08 ± 1.92	5.61 ± 2.06	4.96 ± 1.67
20:5n3 (EPA)	13.63 ± 3.13^a^	10.95 ± 0.47^b^	8.69 ± 1.01^b^	9.64 ± 0.30^b^	9.05 ± 0.36^b^	8.41 ± 0.59^b^
22:6n3 (DHA)	4.52 ± 0.20^a^	4.87 ± 0.21^a^	4.74 ± 0.31^a^	4.05 ± 0.11^b^	3.55 ± 0.06^c^	3.20 ± 0.30^c^
∑saturates	38.23 ± 2.85	38.50 ± 2.09	38.24 ± 0.37	39.85 ± 0.60	38.86 ± 1.26	39.01 ± 0.90
∑monoenes	18.97 ± 1.24	18.83 ± 1.99	18.00 ± 0.98	18.37 ± 1.38	16.59 ± 0.98	17.12 ± 2.57
∑n-6PUFA	19.68 ± 1.92^d^	22.36 ± 1.92^c^	26.80 ± 0.33^a^	24.02 ± 0.53^bc^	26.33 ± 1.14^ab^	27.29 ± 1.51^a^
∑n-3PUFA	23.12 ± 4.86^a^	20.31 ± 1.50^ab^	16.97 ± 1.45^c^	17.77 ± 2.09^c^	18.22 ± 1.82^c^	16.57 ± 1.47^c^
∑n-3lcPUFA	18.16 ± 2.98^a^	15.83 ± 0.67^ab^	13.43 ± 0.67^bc^	13.68 ± 0.32^bc^	12.61 ± 0.30^c^	11.61 ± 0.87^c^
∑n-3/∑n-6	1.20 ± 0.35^a^	0.91 ± 0.01^b^	0.63 ± 0.06^bc^	0.74 ± 0.11^bc^	0.69 ± 0.08^bc^	0.61 ± 0.03^c^

**Notes.**

Values are given as the mean ± standard deviation (*n* = 3). Values with differing superscript letters (a–d) highlight significant differences at *p* ≤ 0.05.

∑saturates = 14:0, 15:0, 16:0, 17:0, 18:0.

∑monoenes = 16:1, 17:1, 18:1n9.

∑n-6PUFA = 18:2n-6, 20:4n-6.

∑n-3PUFA = 18:3n-3, 20:5n-3, 22:6-n-3.

∑n-3lcPUFA = 20:5n-3, 22:6-n-3.

**Table 5:** Fatty acid compositions (% of total fatty acids) of stage II–V ovaries of *M. rosenbergii* fed different experimental diets.

Fatty acids	A0	A1	A2	B0	B1	B2
	2%FO+2%SO (Base diet A)			1.5%FO+2.5%SO (Base diet B)		
	0%ARA	0.4%ARA	0.8%ARA	0%ARA	0.4%ARA	0.8%ARA
**Stage II**						
16:0	13.37 ± 0.59^d^	18.96 ± 0.64^c^	19.49 ± 0.94^bc^	21.20 ± 0.02^a^	20.63 ± 0.36^ab^	19.69 ± 0.77^bc^
18:0	6.47 ± 0.08^d^	7.42 ± 0.23^c^	7.62 ± 0.29^c^	7.92 ± 0.07^bc^	8.34 ± 0.38^ab^	8.72 ± 0.66^a^
18:1n9	18.85 ± 0.07^c^	18.02 ± 0.62^c^	22.15 ± 0.23^a^	20.71 ± 0.19^b^	20.78 ± 0.86^b^	19.14 ± 0.87^c^
18:2n6(LOA)	17.09 ± 0.73^c^	15.69 ± 0.41^d^	18.21 ± 0.92^b^	20.25 ± 0.04^a^	19.06 ± 0.58^b^	18.00 ± 0.23^bc^
20:4n6(ARA)	2.35 ± 0.05^d^	4.12 ± 0.06^b^	4.42 ± 0.11^b^	3.15 ± 0.18^c^	4.47 ± 0.44^b^	5.65 ± 0.32^a^
18:3n3(LNA)	0.56 ± 0.09^d^	1.13 ± 0.08^bc^	1.01 ± 0.09^c^	1.37 ± 0.02^a^	1.21 ± 0.09^b^	1.16 ± 0.08^bc^
20:5n3(EPA)	2.40 ± 0.09^a^	2.34 ± 0.097^ab^	2.32 ± 0.09^ab^	2.35 ± 0.06^ab^	2.07 ± 0.17^b^	1.69 ± 0.02^c^
22:6n3(DHA)	5.26 ± 0.02^a^	5.09 ± 0.22^a^	5.10 ± 0.04^a^	4.61 ± 0.07^b^	4.13 ± 0.14^c^	4.33 ± 0.20^bc^
∑saturates	28.40 ± 0.72^c^	31.20 ± 0.92^b^	31.57 ± 1.41^b^	33.90 ± 0.08^a^	34.32 ± 0.56^a^	34.86 ± 1.17^a^
∑monoenes	20.64 ± 0.15^c^	21.60 ± 0.50^bc^	25.97 ± 1.33^a^	24.77 ± 0.18^a^	24.80 ± 1.06^a^	22.78 ± 0.93^b^
∑n-6PUFA	19.45 ± 0.77^b^	19.81 ± 0.36^b^	22.63 ± 0.92^a^	23.41 ± 0.22^a^	23.53 ± 0.25^a^	23.65 ± 0.11^a^
∑n-3PUFA	8.22 ± 0.09^a^	8.55 ± 0.22^a^	8.43 ± 0.15^a^	8.32 ± 0.14^a^	7.41 ± 0.38^b^	7.18 ± 0.18^b^
∑n-3lcPUFA	7.66 ± 0.12^a^	7.43 ± 0.23^ab^	7.42 ± 0.57^ab^	6.96 ± 0.13^b^	6.20 ± 0.29^c^	6.02 ± 0.21^c^
∑n-3/∑n-6	0.42 ± 0.02^a^	0.43 ± 0.01^a^	0.37 ± 0.02^b^	0.36 ± 0.01^b^	0.31 ± 0.02^c^	0.31 ± 0.01^c^
**Stage III**						
16:0	15.30 ± 0.36^d^	21.51 ± 0.02^a^	21.42 ± 0.16^ab^	21.06 ± 0.04^c^	20.91 ± 0.06^c^	21.12 ± 0.10^bc^
18:0	7.71 ± 0.03^b^	7.02 ± 0.02^d^	7.31 ± 0.04^c^	7.21 ± 0.09^c^	7.22 ± 0.11^c^	8.06 ± 0.09^a^
18:1n9	21.84 ± 0.05^c^	22.34 ± 0.03^b^	22.28 ± 0.04^b^	24.63 ± 0.22^a^	22.15 ± 0.15^bc^	19.58 ± 0.44^d^
18:2n6(LOA)	18.99 ± 0.07^c^	17.59 ± 0.15^d^	19.13 ± 0.35^bc^	19.94 ± 0.07^a^	19.55 ± 0.17^ab^	19.23 ± 0.49^bc^
20:4n6(ARA)	2.93 ± 0.06^d^	4.52 ± 0.25^c^	5.03 ± 0.03^b^	2.84 ± 0.28^d^	5.88 ± 0.12^a^	6.16 ± 0.14^a^
18:3n3(LNA)	1.56 ± 0.11^a^	1.34 ± 0.04^b^	1.53 ± 0.01^a^	1.57 ± 0.02^a^	1.33 ± 0.04^b^	1.26 ± 0.02^b^
20:5n3(EPA)	4.56 ± 0.60^b^	4.56 ± 0.13^b^	5.27 ± 0.17^a^	4.56 ± 0.03^b^	4.66 ± 0.37^b^	3.54 ± 0.24^c^
22:6n3(DHA)	7.03 ± 0.06^a^	6.17 ± 0.07^c^	6.27 ± 0.05^bc^	6.53 ± 0.31^b^	5.43 ± 0.07^d^	4.06 ± 0.11^e^
∑saturates	27.71 ± 0.43^d^	34.43 ± 0.13^c^	34.68 ± 0.22^b^	34.41 ± 0.14^c^	34.94 ± 0.30^ab^	35.36 ± 0.17^a^
∑monoenes	26.72 ± 0.04^bc^	26.82 ± 0.09^b^	26.44 ± 0.06^c^	29.54 ± 0.22^a^	26.49 ± 0.08^bc^	23.44 ± 0.40^d^
∑n-6PUFA	21.92 ± 0.04^d^	22.11 ± 0.14^d^	24.16 ± 0.33^b^	22.78 ± 0.22^c^	25.43 ± 0.28^a^	25.39 ± 0.51^a^
∑n-3PUFA	13.15 ± 0.54^a^	12.07 ± 0.10^b^	13.07 ± 0.21^a^	12.65 ± 0.27^a^	11.42 ± 0.39^c^	8.86 ± 0.15^d^
∑n-3lcPUFA	11.59 ± 0.54^a^	10.73 ± 0.07^b^	11.54 ± 0.21^a^	11.09 ± 0.29^ab^	10.09 ± 0.43^c^	7.60 ± 0.15^d^
∑n-3/∑n-6	0.60 ± 0.03^a^	0.55 ± 0.01^b^	0.54 ± 0.01^b^	0.56 ± 0.01^b^	0.45 ± 0.02^c^	0.35 ± 0.01^d^
**Stage IV**						
16:0	20.44 ± 0.03^a^	20.52 ± 0.14^a^	20.40 ± 0.15^ab^	20.05 ± 0.03^c^	20.22 ± 0.06^bc^	19.74 ± 0.14^d^
18:0	6.98 ± 0.02^a^	6.46 ± 0.12^b^	6.52 ± 0.26^b^	5.86 ± 0.16^c^	6.61 ± 0.11^b^	6.41 ± 0.10^b^
18:1n9	24.23 ± 0.25^b^	21.39 ± 0.20^f^	22.76 ± 0.07^d^	24.86 ± 0.15^a^	21.70 ± 0.17^e^	23.45 ± 0.14^c^
18:2n6(LOA)	20.11 ± 0.13^b^	19.88 ± .04^bc^	18.64 ± 0.12^d^	20.93 ± 0.15^a^	19.45 ± 0.53^c^	21.03 ± 0.18^a^
20:4n6(ARA)	3.02 ± 0.45^e^	5.10 ± 0.12^d^	5.67 ± 0.41^c^	2.46 ± 0.24^f^	6.17 ± 0.32^b^	7.05 ± 0.08^a^
18:3n3(LNA)	1.49 ± 0.01^bc^	1.25 ± 0.03^e^	1.39 ± 0.01^d^	1.72 ± 0.03^a^	1.46 ± 0.04^c^	1.54 ± 0.03^b^
20:5n3(EPA)	3.30 ± 0.17^c^	4.61 ± 0.19^b^	4.60 ± 0.04^b^	4.90 ± 0.10^a^	5.02 ± 0.02^a^	5.10 ± 0.02^a^
22:6n3(DHA)	6.34 ± 0.19	6.06 ± 0.06	6.14 ± 0.05	6.19 ± 0.25	6.06 ± 0.15	6.08 ± 0.10
∑saturates	33.50 ± 0.15^a^	31.28 ± 0.06^b^	31.09 ± 0.09^b^	29.54 ± 0.17^d^	30.69 ± 0.08^c^	30.52 ± 0.17^c^
∑monoenes	27.83 ± 0.13^b^	25.50 ± 0.31^f^	26.93 ± 0.18^d^	29.36 ± 0.17^a^	25.99 ± 0.18^e^	27.25 ± 0.06^c^
∑n-6PUFA	23.13 ± 0.18^d^	24.98 ± 0.07^bc^	24.31 ± 0.54^c^	23.39 ± 0.27^d^	25.62 ± 0.71^b^	28.11 ± 0.24^a^
∑n-3PUFA	11.14 ± 0.29^d^	11.91 ± 0.27^c^	12.13 ± 0.03^bc^	12.81 ± 0.32^a^	12.53 ± 0.14^ab^	12.72 ± 0.06^a^
∑n-3lcPUFA	9.65 ± 0.29^c^	10.66 ± 0.25^b^	10.74 ± 0.03^b^	11.09 ± 0.31^ab^	11.08 ± 0.14^ab^	11.19 ± 0.09^a^
∑n-3/∑n-6	0.48 ± 0.01^bc^	0.48 ± 0.01^c^	0.50 ± 0.01^b^	0.55 ± 0.01^a^	0.49 ± 0.03^bc^	0.45 ± 0.01^d^
**Stage V**						
16:0	21.05 ± 0.13^a^	20.20 ± 0.53^c^	19.13 ± 0.15^d^	21.17 ± 0.06^a^	20.80 ± 0.06^ab^	20.40 ± 0.05^bc^
18:0	4.90 ± 0.15^d^	7.44 ± 0.28^b^	7.20 ± 0.07^b^	6.41 ± 0.05^c^	7.25 ± 0.06^b^	7.77 ± 0.07^a^
18:1n9	15.82 ± 0.11^d^	22.84 ± 0.13^a^	22.41 ± 0.28^b^	21.98 ± 0.21^c^	22.44 ± 0.12^b^	21.96 ± 0.22^c^
18:2n6(LOA)	17.41 ± 0.27^c^	17.15 ± 0.06^c^	16.92 ± 0.68^c^	18.05 ± 0.08^b^	19.28 ± 0.42^a^	19.43 ± 0.15^a^
20:4n6(ARA)	2.14 ± 0.15^e^	4.02 ± 0.08^d^	5.39 ± 0.34^a^	1.99 ± 0.02^e^	4.99 ± 0.02^b^	4.56 ± 0.09^c^
18:3n3(LNA)	1.16 ± 0.04^e^	1.41 ± 0.02^bc^	1.48 ± 0.07^b^	1.64 ± 0.05^a^	1.33 ± 0.03^d^	1.38 ± 0.01^cd^
20:5n3(EPA)	5.25 ± 0.03^b^	4.87 ± 0.13^c^	4.14 ± 0.05^e^	4.56 ± 0.02^d^	5.53 ± 0.16^a^	4.41 ± 0.04^d^
22:6n3(DHA)	6.96 ± 0.03^a^	6.09 ± 0.13^d^	4.55 ± 0.14^e^	6.40 ± 0.04^c^	6.70 ± 0.11^b^	6.01 ± 0.02^d^
∑saturates	30.23 ± 0.23^c^	31.27 ± 0.78^b^	29.71 ± 0.20^c^	31.22 ± 0.07^b^	32.25 ± 0.12^a^	32.00 ± 0.07^a^
∑monoenes	20.60 ± 0.12^c^	26.78 ± 0.12^a^	26.30 ± 0.34^b^	26.86 ± 0.16^a^	26.61 ± 0.11^ab^	26.64 ± 0.22^ab^
∑n-6PUFA	19.55 ± 0.16^d^	21.17 ± 0.13^c^	22.31 ± 0.79^b^	20.04 ± 0.09^d^	24.26 ± 0.41^a^	23.98 ± 0.16^a^
∑n-3PUFA	13.37 ± 0.06^a^	12.37 ± 0.21^b^	10.16 ± 0.21^d^	12.59 ± 0.04^b^	13.57 ± 0.16^a^	11.80 ± 0.03^c^
∑n-3lcPUFA	12.21 ± 0.02^a^	10.96 ± 0.19^b^	8.68 ± 0.18^d^	10.96 ± 0.07^b^	12.23 ± 0.13^a^	10.42 ± 0.03^c^
∑n-3/∑n-6	0.68 ± 0.01^a^	0.58 ± 0.01^c^	0.46 ± 0.01^f^	0.63 ± 0.01^b^	0.56 ± 0.03^d^	0.49 ± 0.01^e^

**Notes.**

Values are given as the mean ± standard deviation (*n* = 3). Values with differing superscript letters (a–e) highlight significant differences at *p* ≤ 0.05.

**Table 6:** Fatty acid compositions (% of total fatty acids) of newly fertilized eggs (OE), mid-stage embryo (BE) and larvae of *M. rosenbergii* fed different experimental diets.

Fatty acids	A0	A1	A2	B0	B1	B2
	2%FO+2%SO (Base diet A)			1.5%FO+2.5%SO (Base diet B)		
	0%ARA	0.4%ARA	0.8%ARA	0%ARA	0.4%ARA	0.8%ARA
**Newly fertilized, orange eggs (OE)**						
16:0	20.47 ± 0.14^bc^	20.97 ± 0.67^abc^	21.45 ± 0.44^ab^	20.40 ± 0.68^c^	20.86 ± 0.41^abc^	21.66 ± 0.27^a^
18:0	6.54 ± 0.13	6.74 ± 0.12	6.60 ± 0.22	6.66 ± 0.18	6.79 ± 0.43	6.78 ± 0.03
18:1n9	21.70 ± 0.17^d^	21.69 ± 0.13^d^	22.19 ± 0.10^c^	22.15 ± 0.18^c^	23.52 ± 0.07^b^	24.37 ± 0.25^a^
18:2n6(LOA)	18.20 ± 0.17^a^	18.02 ± 0.19^a^	17.74 ± 0.10^b^	18.07 ± 0.15^a^	16.56 ± 0.12^c^	16.30 ± 0.06^d^
20:4n6(ARA)	3.82 ± 0.09^c^	4.65 ± 0.14^b^	5.48 ± 0.09^a^	2.67 ± 0.06^d^	5.77 ± 0.15^a^	5.89 ± 0.04^a^
18:3n3(LNA)	0.63 ± 0.03^b^	0.36 ± 0.03^c^	0.65 ± 0.01^b^	0.66 ± 0.05^b^	1.15 ± 0.03^a^	1.14 ± 0.03^a^
20:5n3(EPA)	8.31 ± 0.11^a^	8.59 ± 0.02^a^	8.06 ± 0.04^a^	8.22 ± 0.02^a^	4.15 ± 0.51^b^	4.07 ± 0.08^b^
22:6n3(DHA)	5.33 ± 0.11^b^	5.49 ± 0.05^b^	5.32 ± 0.06^b^	3.39 ± 0.05^c^	6.13 ± 0.16^a^	6.33 ± 0.10^a^
∑saturates	32.38 ± 0.51^ab^	32.85 ± 0.91^a^	32.97 ± 0.39^a^	31.55 ± 0.45^b^	31.40 ± 0.46^b^	32.17 ± 0.34^ab^
∑monoenes	26.67 ± 0.09^d^	26.84 ± 0.16^d^	27.31 ± 0.15^c^	27.29 ± 0.18^c^	29.60 ± 0.09^b^	30.80 ± 0.24^a^
∑n-6PUFA	22.02 ± 0.13^c^	22.67 ± 0.05^b^	23.22 ± 0.12^a^	20.74 ± 0.12^d^	22.33 ± 0.14^bc^	22.20 ± 0.04^c^
∑n-3PUFA	14.27 ± 0.13^a^	14.44 ± 0.50^a^	14.02 ± 0.25^a^	12.27 ± 0.79^b^	11.43 ± 0.39^c^	11.54 ± 0.17^bc^
∑n-3lcPUFA	13.64 ± 0.16^a^	14.08 ± 0.03^a^	13.38 ± 0.02^a^	11.61 ± 0.04^b^	10.27 ± 0.40^c^	10.40 ± 0.18^c^
∑n-3/∑n-6	0.65 ± 0.01^a^	0.64 ± 0.01^ab^	0.60 ± 0.02^bc^	0.59 ± 0.01^c^	0.51 ± 0.02^d^	0.52 ± 0.01^d^
**Mid-stage embryo, brownish eggs (BE)**						
16:0	22.47 ± 0.46^a^	22.10 ± 0.37^ab^	22.06 ± 0.67^ab^	20.05 ± 0.02^d^	21.36 ± 0.29^b^	22.26 ± 0.33^a^
18:0	6.89 ± 0.13^e^	7.98 ± 0.05^c^	8.57 ± 0.05^b^	7.34 ± 0.05^d^	8.71 ± 0.23^b^	8.96 ± 0.33^a^
18:1n9	23.97 ± 0.06^b^	23.59 ± 0.14^b^	24.51 ± 0.13^a^	23.60 ± 0.06^b^	21.54 ± 0.48^c^	21.45 ± 0.05^c^
18:2n6(LOA)	19.02 ± 0.14^b^	18.05 ± 0.26^d^	18.52 ± 0.27^c^	18.92 ± 0.13^b^	19.03 ± 0.09^b^	19.84 ± 0.24^a^
20:4n6(ARA)	3.51 ± 0.07^d^	4.72 ± 0.18^c^	5.36 ± 0.03^b^	1.46 ± 0.17^e^	4.72 ± 0.05^c^	5.77 ± 0.21^a^
18:3n3(LNA)	1.30 ± 0.02^a^	1.23 ± 0.06^b^	1.14 ± 0.03^c^	0.91 ± 0.03^e^	0.96 ± 0.02^e^	1.04 ± 0.02^d^
20:5n3(EPA)	4.59 ± 0.09^b^	4.99 ± 0.06^a^	4.61 ± 0.08^b^	3.48 ± 0.23^d^	3.90 ± 0.05^c^	3.82 ± 0.22^c^
22:6n3(DHA)	5.72 ± 0.11^b^	6.15 ± 0.39^a^	5.22 ± 0.20^c^	4.42 ± 0.16^d^	4.91 ± 0.06^c^	4.45 ± 0.03^d^
∑saturates	34.14 ± 0.35^ab^	34.57 ± 0.38^ab^	34.84 ± 0.72^a^	31.22 ± 0.04^c^	33.82 ± 0.49^b^	34.98 ± 0.45^a^
∑monoenes	28.78 ± 0.17^ab^	28.27 ± 0.19^c^	29.16 ± 0.16^a^	28.47 ± 0.15^bc^	26.27 ± 0.56^d^	26.32 ± 0.05^d^
∑n-6PUFA	22.53 ± 0.21^c^	22.77 ± 0.24^c^	23.88 ± 0.25^b^	20.39 ± 0.04^d^	23.74 ± 0.3	25.61 ± 0.43^a^
∑n-3PUFA	11.60 ± 0.20^b^	12.38 ± 0.38^a^	10.97 ± 0.14^c^	8.81 ± 0.12^f^	9.77 ± 0.02^d^	9.32 ± 0.27^e^
∑n-3lcPUFA	10.31 ± 0.19^b^	11.15 ± 0.33^a^	9.83 ± 0.14^c^	7.90 ± 0.10^f^	8.81 ± 0.04^d^	8.27 ± 0.25^e^
∑n-3/∑n-6	0.51 ± 0.01^b^	0.54 ± 0.02^a^	0.46 ± 0.01^c^	0.43 ± 0.01^d^	0.41 ± 0.01^e^	0.36 ± 0.01^f^
**Larvae**						
16:0	23.69 ± 0.15^c^	21.26 ± 0.16^e^	22.50 ± 0.50^d^	23.47 ± 0.14^c^	24.74 ± 0.08^a^	24.30 ± 0.11^b^
18:0	8.68 ± 0.18^c^	8.69 ± 0.14^c^	9.69 ± 0.15^a^	9.28 ± 0.06^b^	9.58 ± 0.10^a^	9.59 ± 0.06^a^
18:1n9	21.01 ± 0.11^a^	19.27 ± 0.07^d^	19.92 ± 0.21^c^	18.97 ± 0.33^d^	20.11 ± 0.12^c^	20.62 ± 0.23^b^
18:2n6(LOA)	16.70 ± 0.06^b^	15.85 ± 0.06^c^	16.21 ± 0.53^bc^	16.99 ± 0.67^b^	16.38 ± 0.54^bc^	18.01 ± 0.20^a^
20:4n6(ARA)	2.26 ± 0.04^e^	4.12 ± 0.10^c^	5.38 ± 0.06^b^	2.74 ± 0.20^d^	5.34 ± 0.91^b^	6.51 ± 0.15^a^
18:3n3(LNA)	1.08 ± 0.01^a^	0.90 ± 0.05^c^	0.94 ± 0.03^bc^	1.01 ± 0.08^ab^	1.00 ± 0.04^ab^	1.06 ± 0.02^a^
20:5n3(EPA)	5.75 ± 0.13^bc^	5.86 ± 0.16^bc^	5.62 ± 0.27^c^	7.06 ± 0.17^a^	6.31 ± 0.31^b^	5.85 ± 0.04^bc^
22:6n3(DHA)	5.05 ± 0.10^a^	4.57 ± 0.23^b^	4.43 ± 0.04^b^	4.30 ± 0.07^b^	5.24 ± 0.23^a^	5.05 ± 0.12^a^
∑saturates	37.45 ± 0.06^d^	34.31 ± 0.16^e^	37.41 ± 0.30^d^	38.22 ± 0.15^c^	39.75 ± 0.21^a^	39.37 ± 0.09^b^
∑monoenes	25.41 ± 0.18^a^	23.09 ± 0.08^d^	23.92 ± 0.24^b^	23.31 ± 0.37^d^	23.92 ± 0.13^b^	24.56 ± 0.21^b^
∑n-6PUFA	18.96 ± 0.03^d^	19.97 ± 0.12^c^	21.60 ± 0.55^b^	19.74 ± 0.61^c^	21.72 ± 0.46^b^	24.53 ± 0.11^a^
∑n-3PUFA	11.88 ± 0.21^ab^	11.33 ± 0.35^bc^	10.99 ± 0.35^c^	12.38 ± 0.65^a^	12.55 ± 0.35^a^	11.95 ± 0.07^ab^
∑n-3lcPUFA	10.80 ± 0.22^ab^	10.42 ± 0.34^bc^	10.05 ± 0.30^c^	11.37 ± 0.73^a^	11.55 ± 0.37^a^	10.90 ± 0.09^ab^
∑n-3/∑n-6	0.63 ± 0.02^a^	0.57 ± 0.02^b^	0.51 ± 0.01^c^	0.63 ± 0.04^a^	0.58 ± 0.03^b^	0.49 ± 0.01^c^

**Notes.**

Values are given as the mean ± standard deviation (*n* = 3). Values with differing superscript letters (a–e) highlight significant differences at *p* ≤ 0.05.

∑saturates = 14:0, 15:0, 16:0, 17:0, 18:0.

∑monoenes = 16:1, 17:1, 18:1n9.

∑n-6PUFA = 18:2n-6, 20:4n-6.

∑n-3PUFA = 18:3n-3, 20:5n-3, 22:6-n-3.

∑n-3lcPUFA = 20:5n-3, 22:6-n-3.

### Muscle fatty acid profiles

The muscle compositions of mature females (Table 4) typically corresponded to dietary fatty acid compositions (Table 2), but with muscle fatty acids containing increased percentages of total saturates, ARA, and EPA. Meanwhile there were deceased percentages of total monoenes, LOA, LNA and DHA in muscle. For PUFA, except for prawns fed the A0 diet, muscle total n-6 PUFA contents were higher than total n-3 PUFA; the total n-3 to total n-6 PUFA ratios ranged from 0.61 (B2) to 1.20 (A0). The primary fatty acid contributing to the total n-6 PUFA in muscle was LOA (approximately 14–18%). For total n-3 PUFA in muscle, EPA contributed to approximately 10% of total fatty acids while LNA and DHA each contributed to approximately 5%. In contrast to the diets, the muscle EPA contents in all experimental groups were higher than DHA and LNA.

Among experimental treatments, muscle total saturates and total monoenes were not statistically different, but some PUFAs, namely LOA, ARA, EPA and DHA, showed some significant variation. The differences in LOA and DHA in muscle were consistent with the differences of these two fatty acids between the two base diets. Dietary treatment B had a higher muscle LOA level while dietary treatment A had a higher muscle DHA level (*p* < 0.05). Variation of muscle ARA percentages to total fatty acids corresponded to the percentages of ARA added to diets and source of fatty acid in each base diet. Groups fed diets A2 had the highest proportions of muscle ARA followed by B2, A1and B1 while those fed diet A0 and B0 had the lowest muscle ARA level (*p* < 0.05). For n-3 PUFA, muscle EPA was highest in the A0 group (*p* < 0.05) while the remaining treatments showed similar values (*p* > 0.05). The total n-3 PUFA in muscle was highest in prawns fed diets A0 and A1, while the remaining dietary treatments exhibited similar values.

### Ovarian fatty acid profiles

Ovarian fatty acid profiles differed from those of muscle in the contents of saturates, monoenes, LOA, ARA and EPA (Table 5 and Fig. 2A). Saturates were dominant in muscle whereas monoenes were dominant in reproductive tissues. Compared to muscle and diets, ovarian fatty acids contained substantially decreased percentages of total n-3 PUFA. Muscle total n-6 PUFA content was similar to the ovarian level, but proportional contents of individual n-6 PUFA, namely ARA and LOA, differed between the two tissue types. Compared to muscle levels, ovarian ARA was much lower while ovarian LOA was slightly higher. The decreased percentages of total n-3 PUFA was due to substantial reduction of LNA and EPA rather than DHA. Ovarian DHA, on the other hand, was slightly higher than muscle DHA. In contrast to muscle, where EPA was higher than DHA (EPA/DHA >1), ovarian DHA was higher than EPA (EPA/DHA = 0.39–0.9). Ratios of ovarian total n-3 PUFA to total n-6 PUFA were less than 0.68.

Among ovarian tissues, stage II ovaries had a more distinct fatty acid profile than the remaining stages (Fig. 2B); the two components explained approximately 60% of total variation (40% and 19.5% for dimensions 1 and 2, respectively). The fatty acid profiles of stage II ovaries contained much less PUFA than other stages. Depending on the dietary treatment, ovaries II of some treatments contained similar profiles to ovaries III (B2) and V (A2). More mature stages contained similar PUFA, monoene and saturate profiles. As the maturation proceeded, a combination of high proportional contents of saturates, monoenes, LOA, ARA, and EPA were distinctive in ovaries III and IV while that of EPA and DHA were prominent in ovaries at stages IV and V (Table 5 and Fig. 2B).

For most dietary treatments, the contents of total monoenes, LOA, ARA, LNA, EPA and DHA gradually increased from ovarian stage II and peaked at ovarian stages III or IV, then stabilized or slightly decreased at the final stage of maturation (stage V) (Table 5). Ovarian LOA, contributing to a large percentage of the fatty acid profile, increased from 15.69–20.25% in stage II to 18.6–21.03% in stage IV ovaries. Except for treatment B0, ovarian ARA increased from 2.35–5.65% in stage II ovaries to 2.46%–7.08% in stage IV. EPA, on the other hand, sharply increased from stage II to III (from 1.69–2.40% in stage II to 3.54–5.27% in stage III ovaries) and remained at a stable level from stage III to V. DHA increased from 4.13–5.26% in stage II ovaries to 4.06–7.03% in stages III to IV. Much lower levels of ovarian LNA were detected; the levels increased as maturation progressed (from approximately 0.56% in stage II ovaries to 1.72% in stage IV). These LNA levels were similar across all dietary treatments.

For each ovarian maturation stage, females fed different experimental diets varied in their ovarian fatty acid compositions (Table 5), especially between dietary treatments with and without ARA (A0 and B0 vs. A1, A2, B1 and B2). The differences in ovarian fatty acid percentages, especially total saturates, LOA, and the total n-3 lcPUFA, between the two base dietary treatments (A and B), on the other hand, varied by the ovarian developmental stage and were not always obvious. PCA also suggested great variation among treatments within each ovarian stage. Ovaries at stages II and III had the greatest among-treatment variation, with ARA being an important factor. Among treatments, the fatty acid profiles of B2 consistently contained high ARA while that of A0 contained low ARA percentages in all ovarian stages (Fig. 2B).

The variation of ovarian ARA within each ovarian maturation stage corresponded to the dietary ARA supplementation. Compared to dietary treatments without ARA supplementation, groups fed diets with ARA supplementation had higher ovarian ARA levels in all ovarian stages, especially those fed diets B1 and B2 (from 4.47–5.65% in stage II ovary to 6.17–7.05% in stage IV). In all ovarian stages, females fed B2 and B1 typically had the highest ovarian ARA proportional contents, followed by those fed A2 and A1; this relationship between the base diet and ARA retention was opposite to that observed in muscle and diet where treatments A2 and A1 had the highest ARA percentages.

### Egg and larval fatty acid profiles

Egg and larval fatty acid profiles were more similar to the ovarian profiles, especially at stage V ovaries, than to those of muscle (Table 6 and Fig. 2A). However, PCA suggested that fatty acid profiles of egg and larval tissues were distinct based on three principal components (Fig. 2C, only two dimensions are shown); the three dimensions explained 30.6%, 27% and 18.9% of total variation. Based on the first dimension, profiles of BE and OE were different in their proportional contents of total monoenes, ARA, LNA, and DHA (positive correlation to the axis, BE) and EPA (negatively correlated, OE). The second dimension differentiated larval profiles from egg profiles on the basis of total saturates, monoenes, LOA, ARA and DHA, with the larval profiles positively correlated with total saturates, ARA and DHA. The third dimension separated OE and larvae profiles based on EPA (OE) and saturates (larvae). Although there was great variation among treatments within each tissue type, the fatty acid profiles of treatments B1 and B2 in eggs and larvae typically contained high ARA and DHA percentages (Fig. 2B). ARA proportional contents remained high throughout the embryonic development in treatments with ARA supplementation (>4%) whereas the contents decreased as the development proceeded in treatments without ARA addition (Table 6). OE had the greatest variation among treatments.

At the OE stage, the differences in dietary fatty acid levels in the two base diets were consistent only with the differences in total n-3 PUFA and total n-3 lcPUFA (Table 6). Groups fed the base diet A had higher levels of total n-3 PUFA (14.02–14.44%) and total n-3 lcPUFA (13.38–14.08%) in eggs than those fed the base diet B (total n-3PUFA = 11.43–12.27% and total n-3 lcPUFA = 10.27–11.61%) Other classes of fatty acids varied upon dietary treatment. For n-6 PUFA, LOA levels comprised approximately 16-18% of total fatty acids, and were highest in treatments A0, A1, and B0, followed by A2 (*p* < 0.05). Egg ARA was highest in treatments A2, B1, and B2 followed by A1 (*p* < 0.05). For n-3 PUFA, EPA was highest in treatments A0, A1, A2 and B0, but LNA and DHA were highest in B1 and B2 (*p* < 0.05).

At the BE stage, groups fed diet A had higher LNA, EPA, total n-3PUFA, and total n-3 lcPUFA than those fed diet B (*p* < 0.05). For n-6 PUFA, LOA contents were similar across treatments (18.05–19.84%) although treatment B2 showed the highest level at 19.84 ± 0.24%, followed by A0, B0 and B1 (18.92–19.03%). Similar to OE, ARA was highest in treatment B2 followed by A2 and A1/B1 (*p* < 0.05). The variation of total n-6 PUFA among treatments was therefore similar to that of ARA and LOA, with treatment B2 showing the highest values, followed by treatments A2 and B1 (*p* < 0.05). For n-3 PUFA, EPA and DHA percentages were similar across treatments, with A1 showing the highest values (4.99% and 6.15% for EPA and DHA, respectively), and B0 showing the lowest values (3.48% and 4.42% for EPA and DHA, respectively).

In larvae, groups fed the base diet B had higher total saturates in tissue than those fed diet A, with the percentage being highest in treatment B1 (*p* < 0.05). For n-6 PUFA, LOA was highest in treatment B2 and lowest in A1. Similar to egg tissues, larval ARA was highest in treatment B2 followed by A2/B1 (*p* < 0.05). For n-3 PUFA, the total n-3 PUFA and total n-3 lcPUFA were higher in treatments A0, B0, B1 and B2 than those in treatment A2; an opposite pattern was observed in egg tissue. This n-3 PUFA variation was due to high levels of EPA detected in B0 and B1 and high DHA levels detected in A0, B1 and B2.

## Discussion

### Effects of dietary supplementation of Arachidonic acid on *M. rosenbergii* reproduction

Our research highlighted the importance of ARA in females’ reproduction, especially for traits relevant to ovarian maturation and oogenesis, in *M. rosenbergii*. Our results strongly suggested that enriching with *Mortierella alpine*-derived ARA at 0.4 and 0.8% of ingredient weight, regardless of the base diet, greatly improved females’ GSI, HSI, egg clutch weight, and fecundity compared to diets without ARA supplements. These supplementation levels resulted in the proportional contents of ARA of 5.74 ± 0.12% (B1) to 9.43 ± 0.28% (A2) in diets. The basal dietary ARA levels were approximately 3% in treatments without ARA supplement. The enhanced reproduction in *M. rosenbergii* was similar to that of tank domesticated *P. monodon* broodstock fed ARA-supplemented diet (Coman et al., 2011). The experimental diet containing 5.8% ARA of total fatty acid can enhance cumulative percentage of female *P. monodon* spawning, number of spawnings per female and eggs per female as well as enhance hatchability of larvae compared to the control diet (no ARA supplement, 1.1% ARA). These positive effects of dietary ARA supplementation on *M. rosenbergii* reproduction and embryonic development may link to an ARA role as a precursor to prostaglandins (PGs).

ARA is a precursor of PGE2 and PGF2_*α*_ in the cyclooxygenase pathway. PGE2 and PGF2_*α*_ are important signaling molecules in several reproductive functions in crustaceans. The roles of PGE2 have been more extensively examined and demonstrated in several shrimp species, including *M. rosenbergii*. PGE2 is associated with oocyte maturation (e.g., *P. semisulcatus;* Ravid et al., 1999; *M. rosenbergii*, Sumpownon et al., 2015), vitellogenesis (e.g., *P.japonicus;* Tahara & Yano, 2004; *M. rosenbergii*, Sumpownon et al., 2015) and spawning (*M. rosenbergii,* Spaziani, Hinsch & Edwards, 1993; Spaziani, Hinsch & Edwards, 1995). PGF2_*α*_, on the other hand, has been recognized for its importance in ovarian maturation in a few crustaceans, including a crayfish *Procambarus paeninsulanus*, and Panaeid marine shrimp (*P. japonicus*, Tahara & Yano, 2004; *P. monodon*, Wimuttisuk et al., 2013). However, the importance of PGF2_*α*_ may have been underappreciated. Wimuttisuk et al. (2013) detected a much higher level of PGF2_*α*_ than PGE2 in *P. monodon* ovaries. For *M. rosenbergii*, it is not clear whether PGF2_*α*_ is also significant.

In *M. rosenbergii*, the biosynthesis of PGE2 occurs in the ovary and the PGE2 levels are high during early ovarian stages compared to more mature ovaries (Sumpownon et al., 2015). PGE2 and key relevant enzymes, namely cycloxygenase1 and PGE synthase, were present in cytoplasm of previtellogenic oocytes. By injecting 10^−7^–10^−9^ mol/prawn of PGE2 into individuals at ovarian stage I, Sumpownon et al. (2015) found that PGE2 stimulated the ovarian maturation by shortening the length of ovarian cycles as well as enhancing oocyte proliferation and vitellogenin production in hemolymph. Similar to other shrimp species (e.g., Coman et al., 2011; Glencross, 2009 and reference therein), the need for PGE2 production in ovaries of *M. rosenbergii* was well reflected by preferential retention of ARA in ovarian tissues throughout maturation. In most dietary treatments, we observed a peak ARA proportional content in the premature and maturing ovaries. This retention pattern was consistent with that observed in wild *M. rosenbergii* females by Cavalli et al. (2001). It is interesting to note that in dietary treatments with ARA supplementation, the ovarian ARA remained at a high proportion even at the mature stage (stage V; approximately 4–5% of total fatty acids). Based on the variation of lipid classes in midgut gland and ovaries of wild *M. rosenbergii* females, Cavalli et al. (2001) suggested prawns utilized lipids from immediate ingestion rather than from those stored in the midgut gland.

Because larval fatty acid profiles are partially controlled by maternal diets (De Caluwe, Lavens & Sorgeloos, 1995; Glencross, 2009), it is not surprising that this study observed higher levels of ARA in eggs and newly hatched larvae in females fed ARA-supplemented diets than those fed diets without ARA supplementation. It also possible that enhanced dietary ARA continued to be reserved in the mature ovaries, eggs and larvae for the production of PGE2 and for energy during spawning, and embryogenesis as well as for assimilation into structural components of embryonic and larval tissues. However, the hatchability and larval quality was also dependent on the availability of another n-6 PUFA, LOA.

We detected much higher levels of ovarian and egg ARA in the females fed diets supplementing ARA (A1, A2, B1 and B2) than those detected in mature ovaries and eggs of wild *M. rosenbergii*. Cavalli et al. (2001) and Balamurugan, Mariappan & Balasundaram (2015) reported the ARA level in mature ovaries of wild *M. rosenbergii* at approximately 2% of total fatty acids, similar to that observed in the dietary treatments without ARA addition. However, the ovarian ARA levels observed in the ARA supplemented dietary treatments (approximately 4–5% of total fatty acids in ovary stage V and eggs) were comparable to those detected in mature ovaries and eggs of wild *P. monodon* (approximately 5–7% of total fatty acids, (Huang et al., 2008)). This disparity of ARA levels may have been a consequence of an inadequate level of ARA in natural diets of *M. rosenbergii* broodstock (freshwater-based diets) and our base diets (diets A0 and B0). Our findings have an important implication for maturation diets for domesticated broodstock of *M. rosenbergii* in that the broodstock of this species requires ARA levels as high as marine shrimp even though their natural diets are derived mainly from freshwater systems, which may be lacking ARA. Formulating maturation diets from observations in the wild may not optimize the prawn’s ARA requirements. We did not detect negative effects of supplementing ARA at 0.8% (9.43 and 8.33%) of total fatty acids in diets A2 and B2, respectively).

### Positive synergetic effects of enhanced dietary ARA and LOA on females’ reproductive performance, egg hatchability and larval quality

In addition to ARA, we detected a positive synergistic effect of enhanced dietary ARA and LOA on females’ reproductive performance, egg hatchability and larval quality (i.e., low salinity tolerance). The dietary treatments B1 and B2, containing 5.7–8.33% ARA and 21.78–22.49 % LOA of total fatty acid, led to higher reproductive performance of *M. rosenbergii* than treatments A1 and A2, containing 6.17–9.43% ARA and 14.85–13.35% LOA. Positive effects of dietary LOA levels on reproductive performance of broodstock and larval quality observed in this study were consistent with those observed by Cavalli, Lavens & Sorgeloos (1999) for *M. rosenbergii* and Ribeiro et al. (2012) for *M. amazonicum*. Ribeiro et al. (2012) found that the dietary treatment with approximately 12 mg g^−1^ DW LOA could improve fecundity of mature female *M. amazonicum*.

Although some authors speculated that LOA may serve as a raw material for the synthesis of ARA (Reigh & Stickney, 1989), some empirical evidence including our results have been inconclusive. Our results did not support this speculation. The base diet containing higher LOA (20.39–22.49% of total fatty acid) without ARA addition (diet B0) did not improve reproductive performance nor enhance ARA accumulation in tissue compared to the base diet with lower LOA, without ARA addition (diet A0). Also, in another *Macrobrachium* species, *M. borllii*, Gonzalez-Baró & Pollero (1998) did not observe the ARA and EPA production from LOA and LNA. Because it is a major fatty acid in all tissues, LOA may have provided additional energy required during energy-intensive developmental stages, such as oogenesis, vittellogenesis, embryogenesis, spawning, hatching and larval development.

LOA contributed to a large percentage of total fatty acids in all tissue types, suggesting its importance as an energy source and as cellular structural components for *M. rosenbergii*. When the dietary LOA was present at a higher percentage (as in diets B1 and B2), LOA was utilized during muscle tissue formation, ovarian maturation and early embryogenesis. On the other hand, dietary LOA available at a slightly lower level (as in diets A1 and A2) was mostly retained and accumulated in muscle and ovarian tissues. In addition, females fed diets with higher LOA tended to retain higher proportions of LOA in ovaries and larvae. LOA may also have played a role in hatching as proportional contents of LOA in larvae were slightly lower than that of mid-stage embryos.

Eggs of females fed diets with enhanced dietary ARA and LOA also had higher hatchability and resulted in higher numbers of larvae than those of other dietary treatments. Also, larvae from these treatments were more tolerant to low salinity stress. The results suggested the importance of both fatty acids in yolk deposition, embryogenesis and possibly larval osmoregulation. A combination of enriched ARA and LOA in diets may have facilitated the accumulations of other important fatty acids, especially DHA, in eggs and larvae despite the lower n-3 lcPUFA available in diets. Having adequate energy required by these energetically expensive life stages, especially from fertilized eggs (containing mainly yolk) to advanced embryo and larvae (involving organ formation) (Habashy, Sharshar & Hassan, 2012; Yao et al., 2006) may allow for efficient utilization of n3-PUFA, especially DHA, required for tissue and organ formation in embryos and larvae. Larvae will continue to rely on yolk for nutrients during the first larval stage, after which they will need an external food source (Anger, 2001).

### Distribution of fatty acids among tissues

The patterns of fatty acid distribution among various tissues suggested that different fatty acids have different functions. Saturates and monoenes were major fatty acid groups in diets and all tissue types of the experimental animals (each class contributed to more than 20% of total fatty acids in each tissue type). The dominance of these fatty acid classes in muscle, ovarian and egg tissues were similar to those observed in wild and farmed *M. rosenbergii* (wild prawn: Cavalli et al., 2001 (muscle, ovaries and egg); farmed prawn: Balamurugan, Mariappan & Balasundaram, 2015 (ovaries and egg), Li, Sinclair & Li, 2011 (muscle), Bragagnolo & Rodriguez-Amaya, 2001 (muscle), Cavalli, Lavens & Sorgeloos, 1999 (muscle, ovaries and egg), Chanmugam et al., 1983 (muscle)) and other marine shrimp species (*P. monodon,* Huang et al., 2008) (ovaries and egg, farmed shrimp), (O’Leary & Matthews, 1990) (muscle, wild and farmed shrimp); *P. chinensis*, Ji & Xu, 1992; *L. vannamei*, Wouter et al., 2001; other wild shrimp species, Li, Sinclair & Li, 2011) although ovarian tissue of farmed *M. rosenbergii* analyzed in this and other studies tended to contain higher proportions of these fatty acid classes compared to those of marine shrimp. These classes of fatty acids usually serve as a main source of energy for growth (Wehrtmann & Graeve, 1998), ovarian development (Harrison, 1990), embryogenesis (Clarke, Brown & Holmes, 1990; Yao et al., 2006) and early larval development (Roustaian et al., 1999; Yao et al., 2006) as they are typically preferred substrates for b-oxidation reactions (Henderson, 1996).

The pattern of LOA, EPA and DHA accumulation and utilization observed in our study may be reflective of fatty acid requirements specific to this diadromous *M. rosenbergii*. Compared to marine shrimp species, *M. rosenbergii* tends to accumulate higher LOA, comparable EPA, but less DHA in the muscle tissue while it accumulates comparable LOA, but less n-3 lcPUFA in reproductive tissues. Tissues of *M. rosenbergii* adults from natural environments tended to contain higher amounts of LOA (approximately 8.8–16% of total fatty acids in muscle tissue and almost 20% in ovarian and egg tissues) compared to marine shrimp species (approximately 1.3–11.4% of total fatty acids in muscle tissue and 10% in egg tissue) (Chanmugam et al., 1983; Bragagnolo & Rodriguez-Amaya, 2001; Cavalli et al., 2001; Huang et al., 2008; Li, Sinclair & Li, 2011). Marine shrimp species have much higher n-3 lcPUFA, especially DHA content in muscle, ovaries and eggs (approximately 9.1–15.2, 21.7, and 35.8 %) of total fatty acids in muscle, ovaries and eggs, respectively; (Li, Sinclair & Li, 2011; Huang et al., 2008) compared to *M. rosenbergii* (3.8–4.8, 2.5–4.2, and 2.6 % of total fatty acids in muscle, ovaries and eggs, respectively; Cavalli et al., 2001; Balamurugan, Mariappan & Balasundaram, 2015). In freshwater crustaceans, n-6 PUFA appears to be as effective as n-3 PUFA as an energy source (D’Abramo & Sheen, 1993). This n-6 PUFA requirement in *M. rosenbergii* may be due to its diadromous nature. The natural diet of prawns during the growth phase contains components from terrestrial sources, rich in shorter chain PUFAs (C18).

##  Supplemental Information

10.7717/peerj.2735/supp-1 Data S1 Reproductive performance and larval quality of *M. rosenbergii* fed different experimental diets

10.7717/peerj.2735/supp-2 Data S2 Fatty acid compositions (% of total fatty acid) of experimental diets, tissues, eggs and larvae of *M. rosenbergii.*

## Additional Information and Declarations

### Data Availability

The following information was supplied regarding data availability: The raw data has been supplied as a Supplementary File.
